# The functional cancer map: A systems-level synopsis of genetic deregulation in cancer

**DOI:** 10.1186/1755-8794-4-53

**Published:** 2011-06-30

**Authors:** Markus Krupp, Thorsten Maass, Jens U Marquardt, Frank Staib, Tobias Bauer, Rainer König, Stefan Biesterfeld, Peter R Galle, Achim Tresch, Andreas Teufel

**Affiliations:** 1 Department of Medicine I, Johannes Gutenberg University, Mainz, Germany; 2 Institut of Pharmacy and Molecular Biotechnology, Bioquant, University of Heidelberg, INF 267, 69120 Heidelberg, Germany; 3 Institute for Pathology, Johannes Gutenberg University, Mainz, Germany, and Department of Cytopathology, Heinrich Heine University, Düsseldorf, Germany; 4 Gene Center Munich, Department of Chemistry and Biochemistry, Ludwig-Maximilians-University, Munich, Germany

**Keywords:** cancer, systems biology, prognostic marker, microarray, bioinformatics

## Abstract

**Background:**

Cancer cells are characterized by massive dysegulation of physiological cell functions with considerable disruption of transcriptional regulation. Genome-wide transcriptome profiling can be utilized for early detection and molecular classification of cancers. Accurate discrimination of functionally different tumor types may help to guide selection of targeted therapy in translational research. Concise grouping of tumor types in cancer maps according to their molecular profile may further be helpful for the development of new therapeutic modalities or open new avenues for already established therapies.

**Methods:**

Complete available human tumor data of the Stanford Microarray Database was downloaded and filtered for relevance, adequacy and reliability. A total of 649 tumor samples from more than 1400 experiments and 58 different tissues were analyzed. Next, a method to score deregulation of KEGG pathway maps in different tumor entities was established, which was then used to convert hundreds of gene expression profiles into corresponding tumor-specific pathway activity profiles. Based on the latter, we defined a measure for functional similarity between tumor entities, which yielded to phylogeny of tumors.

**Results:**

We provide a comprehensive, easy-to-interpret functional cancer map that characterizes tumor types with respect to their biological and functional behavior. Consistently, multiple pathways commonly associated with tumor progression were revealed as common features in the majority of the tumors. However, several pathways previously not linked to carcinogenesis were identified in multiple cancers suggesting an essential role of these pathways in cancer biology. Among these pathways were 'ECM-receptor interaction', 'Complement and Coagulation cascades', and 'PPAR signaling pathway'.

**Conclusion:**

The functional cancer map provides a systematic view on molecular similarities across different cancers by comparing tumors on the level of pathway activity. This work resulted in identification of novel superimposed functional pathways potentially linked to cancer biology. Therefore, our work may serve as a starting point for rationalizing combination of tumor therapeutics as well as for expanding the application of well-established targeted tumor therapies.

## Background

Cancer is one of the leading causes of death [1]. If segregated by age, cancer, has even surpassed the mortality of heart diseases and has become the deadliest disease in the world, with 12.7 million new cases and 7.6 million deaths worldwide [2]. Furthermore, due to the steadily increase in aging and expansion of the world's population an ongoing increase also in the burden of cancer is predicted for the next decades [2]. Nevertheless, since the year 1990, cancer death rate continuously declined despite increasing numbers of new cancer cases [1], indicating the improvements in diagnostics and development of novel more effective therapies. Therein gene expression profiling made a considerable contribution. This technology can be used to classify diverse types of tumors [3,4] and predict outcome of patients [5-7] as well as response to chemotherapies [8,9]. Several studies utilized global transcriptome and genome profiling to identify phenotypically similar cancers [10-12]. Segal et al. successfully introduced a module map for cancers [11], consisting of gene sets that act in concert to carry out specific function. More recently, a meta-analyses tool for different cancer tissues was established [10]. All of these studies revealed associations between molecular profiles and biological behavior of different tumors, without a detailed integration of regulated genes and signaling pathways. Utilization of these functional associations to place global transcriptome data in a biological context might significantly improve the limited knowledge about common molecular changes across different tumor types.

Here, we employed an extensive functional genomics approach to analyze gene expression profiles of the complete human cancer related microarray datasets from the Stanford Microarray Database [13]. Our aim was to develop a functional cancer map focusing on signaling and metabolic events across multiple cancer types to provide a deeper understanding of common and distinct molecular mechanisms leading to carcinogenesis. This novel approach provides a systematic and unbiased view on cancer development by investigating signaling pathways and functional networks in an easy-to-interpret way. In addition, we demonstrate tumor phylogeny based on semantic aggregated profiles by identifying tumor mechanisms of individual tumor entities from publically available gene expression data. Our analysis revealed important signaling pathways commonly dysregulated in several tumor entities. These results could serve as a starting point for the efficient combination of tumor therapeutics as well as for expanding the application area of well-established tumor therapies.

## Methods

### Gene expression data

Gene expression data were obtained from microarray experiments, downloaded from the Stanford Microarray Database [13]. Ignoring cell line and time course experiments, we collected a dataset of 1402 human two-color cDNA microarrays corresponding to 58 discrete tissues types and about 15000 individual genes. Screening for tumor relevant information in our dataset resulted in 836 tumor arrays, which could be assigned to 33 tumor tissue types defined as tumor entities in our analysis. Additionally 16 tumor classes were allocated to tumor entities representing the tumor differentiation and developmental origin of the tissue, e.g. tumor entities 'Prostate Tumor, Grade T2b', 'Prostate Tumor, Grade T3a' and 'Prostate Tumor, Grade 3b' were assigned to the tumor class 'Prostate'. Downloaded microarray experiments had been analyzed against diverse human controls [13]. In order to avoid divergence due to diverse control data sets, we filtered all tumor classes for common applied controls used in the respective microarray experiments. We always used those data with the largest number of individual datasets with the same one control per tumor class for further evaluation. All other data were discarded. Finally our dataset was reduced to 649 microarrays belonging to 28 tumor entities respectively 16 tumor classes, which were analyzed with the R Project statistical software [14] to identify differentially expressed genes as follows:

Initially the raw data were loaded into R, invalid cDNA probes removed (e.g. flag 'contaminated' or 'failed') and a background correction was applied. After the calculation of the log2 fold changes relative to the control RNA the quantile normalization was applied to each experiment. In all these initial steps we made use of the 'limma' R package [15]. Next, we calculated the variance as well as mean for each gene within the corresponding tumor entities. Moreover, the pooled variance for each gene was calculated to identify global outliers. Pooled variance was defined as weighted mean of the variances within each tumor entity and weighted by the relative sizes of the respective entity. Genes (426 in total) with a pooled variance of all genes greater than the third interquartile range of the pooled variance (3iQR = 0.038) were defined as outliers and excluded from further analysis, to ensure the comparability and correct technical bias due to heterogeneity of the dataset. Additionally, tumor entity specific outliers were eliminated by considering those genes whose variance were greater than the 3iQR of the corresponding tumor entity; depending on tumor entity between 364 and 1053 genes were excluded from the dataset.

**Figure 1:**
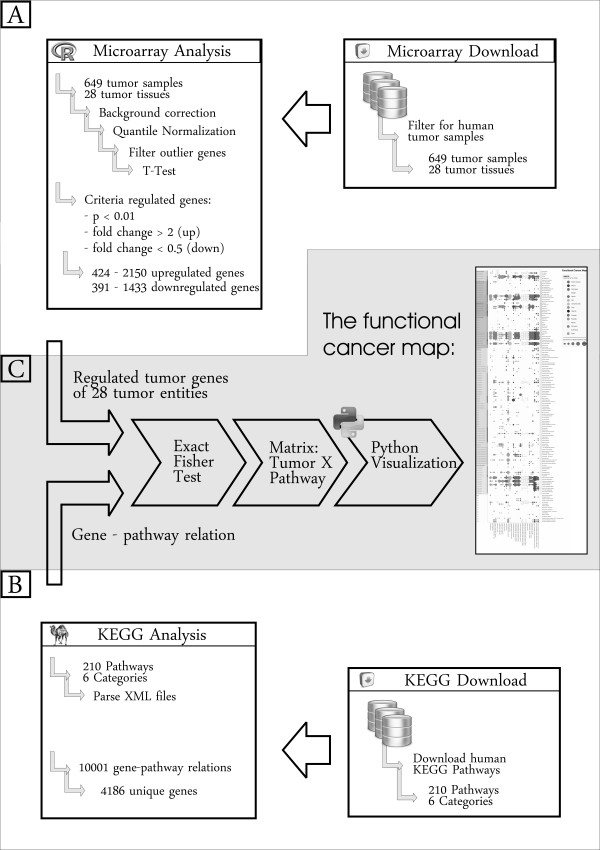
**Schematic drawing of the workflow** - a) gene expression data, b) functional data and c) the functional cancer map.

A gene was considered upregulated (respectively downregulated) if it had a M-value > 1.0 (resp. < -1.0) and a p-value smaller than 0.01 in a one sided one-sample t-test. This procedure defined between 391 and 1433 up- and between 424 and 2150 downregulated tumor associated genes (figure 1a). Note: Although the p-values produced this way are merely used as a score and are not interpreted as type-1 error rates, it might be interesting to assess the significance of the individual tests. We therefore performed an FDR correction using "p.adjust" and provided the list of multiple testing corrected p-values in the additional files (Additional file 1).

### Functional data

Metabolic reaction and signaling event information were acquired from the PATHWAY database of the Kyoto Encyclopedia of Genes and Genomes (KEGG) [16]. These pathway maps and pathway modules are categorized into metabolism, genetic information processing, environmental information processing, cellular process, organismal systems and human diseases in several organisms. Our analysis was based on the KEGG v47 and comprises 210 human pathway maps in XML file format corresponding to 6 categories and 32 subcategories. The data are publically available and can be accessed from the FTP ftp://ftp.genome.jp/pub/. The KEGG pathway data were parsed by a Perl [17] script using the XML::simple module, resulting in 10001 gene-pathway relations belonging to 208 pathway maps and 4186 unique genes (figure 1b).

### Integration of gene expression and functional data

For each tumor entity - KEGG pathway pair, we tested whether the set of genes that are differentially expressed in the given tumor entity is enriched in the set of genes belonging to the given KEGG pathway. Testing was performed using an one-sided exact Fisher test. This resulted in a 28 tumor entities × 208 KEGG pathways matrix containing the p-values of each test.

**Figure 2:**
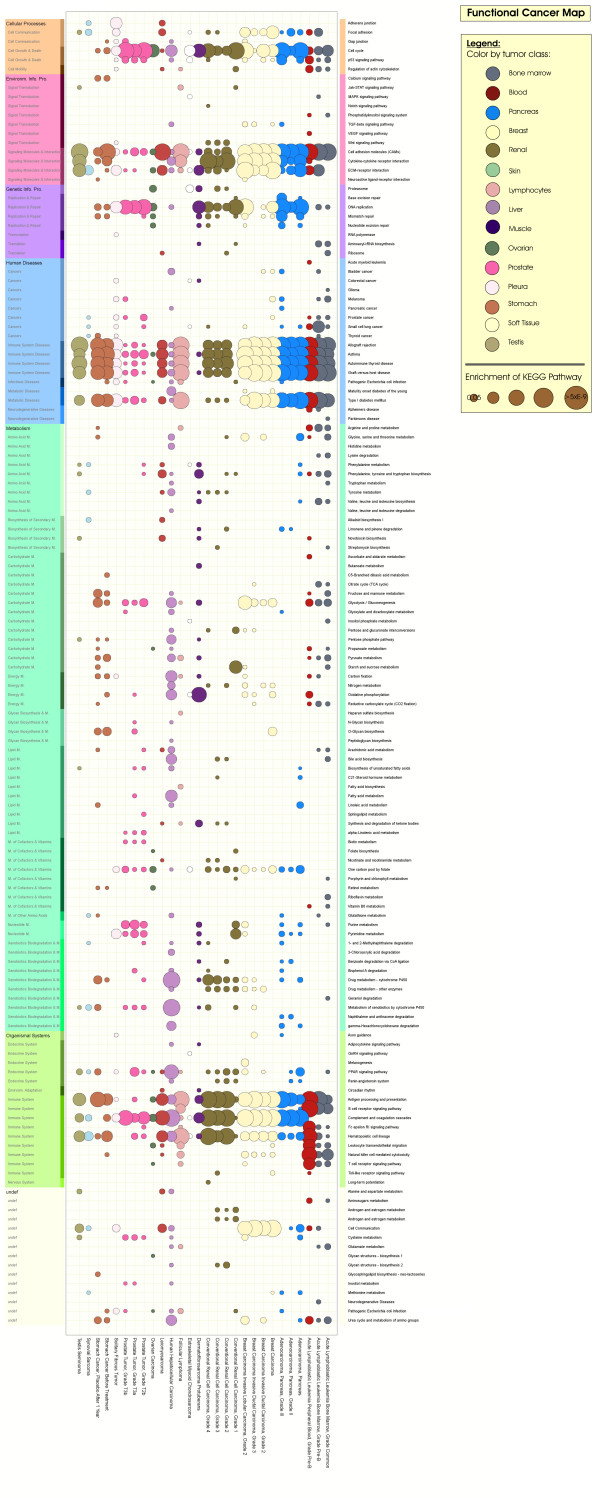
**Functional cancer map: Functional expression profile of significant enriched KEGG pathway maps across 28 tumor entities assigned to 16 tumor classes**. The significance of a tumor entity to a KEGG pathway map is symbolized by the diameter of the spot, the greater the diameter the more significant enriched is the corresponding tumor entity - KEGG pathway map relation. The color of the spot gives information on the tumor entity and its belonging tumor class. Information on the KEGG classification schema is supported by the color gradient on the left side.

To get a better overview, we implemented a Python [18] script, using the PIL module, to visualize significant enriched KEGG pathway maps (p < 0.05) within the tumor entities into a clear structured functional cancer map (figure 1c) (figure 2). The same procedure as described here for the set of all differentially expressed genes can be applied to the set of upregulated resp. downregulated genes separately. The corresponding functional cancer maps are given in the additional files (Additional file 2, Additional file 3). Note: A separately generated functional cancer map without prior filtering for outlier genes (pooled variance and tissue specific variance) was added to the additional files (Additional file 4).

### Literature Mining

Using the web-based tool PubMatrix [19] for literature mining of PubMed [20], we analyzed our significantly enriched KEGG pathway maps against an assortment of tumor associated terms to identify pathways previously mentioned in context to cancer. Tumor associated keywords used in the PubMatrix searches included "Neoplasm", "Tumors", "Tumor", "Benign Neoplasms", "Neoplasms, Benign", "Benign Neoplasm", "Neoplasm, Benign", "Cancer", "Cancers" and "neoplasms[mesh]". PubMatrix is limited to 10000 results for each search and modifier terms. All search results can be found in the additional files (Additional files 5).

### Calculation of tumor phylogeny

In a second step, we used the binarized functional cancer map (1 respectively 0 representing significant resp. non-significant entries) to calculate a similarity score between tumor entities. The similarity between two tumor entities T1 and T2, was quantified by calculating the Cohen's Kappa Coefficient [21] of corresponding columns in the binarized functional cancer map. This coefficient is a statistical measure of agreement for qualitative items.

**Figure 3:**
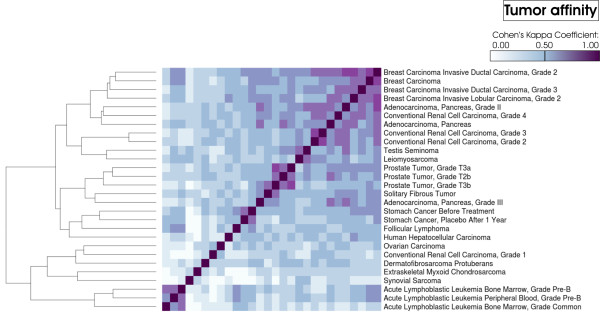
**Tumor phylogeny: Calculation of the tumor phylogeny was done by applying the Cohen's Kappa Coefficient to the binarized functional cancer map, tumor entities - KEGG pathway map relations respectfully**.

We calculated the quadratic agreement matrix among the tumor entities. The result was visualized with the R function heatmap [22]. Euclidean distance average linkage hierarchical clustering [23] was chosen for the dendrogram included in the plot (figure 3). Visualizations of the tumor similarity matrices with respect to upregulated and downregulated genes are given in the additional files (Additional file 6, Additional file 7).

## Results

### Gene expression data

In order to investigate individual and conserved signaling and metabolic events in tumor progression, we analyzed a dataset of 649 human two-color cDNA microarray tumor samples obtained from the Stanford Microarray Database [13]. These samples covered 28 different tumor entities which were further categorized into 16 tumor classes (table1). Analyzing these gene expression profiles revealed a total of 10385 genes differentially expressed in at least one of the tumor entities. Among highly conserved differentially expressed genes were apolipoprotein (*APOH*), previously not mentioned in context with cancer, T cell receptor beta constant 1 (*TRBC1*) a widely undescribed gene and also not associated in context with cancer and alpha-fetoprotein (*AFP*), a major plasma protein reported to be associated with several carcinomas [24-26]. Furthermore Orosomucoid 2 (*ORM2*) another plasma protein whose specific function has not yet been determined and not linked to carcinogenesis was found to be regulated in 25 tumor tissues. Furthermore, two genes were identified to be regulated in 24 tumor tissues, inter-alpha (globulin) inhibitor H2 (*ITIH2*), a gene involved in extracellular matrix stabilization and related to prevention of tumor metastasis [27-29] and the functionally undefined gene *KIAA0101 *which is associated with hepatocellular and thyroid carcinoma [30,31].

**Table 1:** The tumor sample distribution of the 649 microarrays with corresponding controls among the tumor classes and tumor entities used in our analysis

Tumor class	Tumor entity	Analyzed Controls	Number of Samples
Blood	Acute Lymphoblastic Leukemia Peripheral Blood, Grade Pre-B	Stratagene	9
Bone marrow	Acute Lymphoblastic Leukemia Bone Marrow, Grade Common	Stratagene	35
Bone marrow	Acute Lymphoblastic Leukemia Bone Marrow, Grade Pre-B	Stratagene	33
Bone	Extraskeletal Myxoid Chondrosarcoma	CRH	10
Breast	Breast Carcinoma	Stratagene	94
Breast	Breast Carcinoma Invasive Ductal Carcinoma, Grade 2	Stratagene	31
Breast	Breast Carcinoma Invasive Ductal Carcinoma, Grade 3	Stratagene	19
Breast	Breast Carcinoma Invasive Lobular Carcinoma, Grade 2	Stratagene	19
Liver	Human Hepatocellular Carcinoma	Stratagene	19
Lymhpocytes	Follicular Lymphoma	CRG	24
Mixture	Solitary Fibrous Tumor	CRG	7
Muscle	Leiomyosarcoma	CRG	7
Ovarian	Ovarian Carcinoma	CRD	39
Pancreas	Adenocarcinoma, Pancreas	CRH	10
Pancreas	Adenocarcinoma, Pancreas, Grade II	CRH	5
Pancreas	Adenocarcinoma, Pancreas, Grade III	CRH	6
Prostate	Prostate Tumor, Grade T2b	Stratagene	23
Prostate	Prostate Tumor, Grade T3a	Stratagene	19
Prostate	Prostate Tumor, Grade T3b	Stratagene	9
Renal	Conventional Renal Cell Carcinoma, Grade 1	Stratagene	9
Renal	Conventional Renal Cell Carcinoma, Grade 2	Stratagene	34
Renal	Conventional Renal Cell Carcinoma, Grade 3	Stratagene	94
Renal	Conventional Renal Cell Carcinoma, Grade 4	Stratagene	40
Skin	Dermatofibrosarcoma Protuberans	CRG	6
Soft Tissue	Synovial Sarcoma	CRG	5
Stomach	Stomach Cancer Before Treatment	CRF	14
Stomach	Stomach Cancer, Placebo After 1 Year	CRF	13
Testis	Testis Seminoma	CRG	16

### Exposing functional mechanisms

Focusing on deregulated genetic signaling events across multiple tumors as well as metabolic events in tumor progression, we analyzed the differentially expressed genes in context of the functional categories as well as signaling and metabolic pathway maps provided by the Kyoto Encyclopedia of Genes and Genomes (KEGG) [16]. After assigning the KEGG data to gene expression data, a total of 141 KEGG pathway maps were significantly enriched and revealed four well conserved subcategories among the tumor entities: 'Cell Growth and Death', 'Signaling Molecules and Interactions', 'Immune System' and 'Immune System Diseases'. The most highly conserved KEGG pathway was 'Cell adhesion molecules (CAMs)' (p value ranging between 5.58e-13 and 0.01) which is significantly enriched in 26 tumor entities, followed by the KEGG pathway map 'Asthma' (1.37e-12 - 0.01), 'Cell cycle' (1.42e-09 - 0.02) and 'Hematopoietic cell lineage' (1.07e-7 - 0.04) in 25 tumor entities. 'Antigen processing and presentation' (2.59e10 - 0.02), 'ECM-receptor interaction' (7.11e-9 - 0.04), 'Complement and coagulation cascades' (1.66e-15 - 0.03) and 'Graft-versus-host disease' (9.37e-10 - 0.04) KEGG pathway maps were found to be significantly enriched in 24 tumor entities. As a result, we generated a functional cancer map to visualize this large amount of multiple functional tumor data into a clearly structured compendium (figure 2).

### Literature Mining

To evaluate the significantly enriched KEGG pathway maps in context of published literature to tumor progression already described and mostly unknown we utilized the web-based tool PubMatrix [19]. The 'Cell cycle' pathway was the most cited pathway with focus on tumor progression displaying more than 1000000 search results per modifier terms in total. Furthermore, 'Melanoma' and 'Acute myeloid leukemia' with 718282 respectively 493765 tumor associated publications were ranked at the top. Taking into count highly enriched KEGG pathway maps, among the KEGG pathway map 'Cell cycle' in KEGG class 'Cellular Processes', the 'Nucleotide excision repair' (174594) in 'Genetic Information Processing' as well as 'Wnt signaling pathway' (21699) in 'Environmental Information Processing' were highly linked to tumor publications. In contrast, highly enriched KEGG pathway maps 'ECM-receptor interaction' (4), the 'Complement and Coagulation cascades' (35) and the 'PPAR signaling pathway' (238) were mostly undescribed. The complete search results can be found in the additional files (Additional file 5).

### Exposing tumor phylogeny

Further use of the functional cancer map was made to calculate tumor phylogeny by laying down new starting points for the efficient combination of tumor therapeutics as well as for expanding the application area of well-established tumor therapies. We postulated that tumor entities with comparable significantly enriched KEGG pathway maps demonstrate comparable activation of biochemical processes and, thus, biochemical behavior. As a measure of closer or distant relationship between two tumor entities we applied the Cohen's Kappa Coefficient [21] to the binarized functional cancer map data. Analyzing phylogeny between diverse tumor entities, highest concordance was found between tumor entities within the same tissue class. The tumor tissue 'Conventional Renal Cell Carcinoma, Grade 2' and 'Conventional Renal Cell Carcinoma, Grade 3' showed the highest concordance κ = 0.77, followed by 'Breast Carcinoma' and 'Breast Carcinoma Invasive Ductal Carcinoma, Grade 2' (κ = 0.74) and 'Breast Carcinoma Invasive Ductal Carcinoma, Grade 2' and 'Breast Carcinoma Invasive Ductal Carcinoma, Grade 2' (κ = 0.70). Examining the tumor entities belonging to different tumor classes, 'Breast Carcinoma Invasive Ductal Carcinoma, Grade 2' and 'Adenocarcinoma, Pancreas, Grade II' (κ = 0.69) displayed the most functional similarity. Also, high similarity in biological behavior was found between 'Conventional Renal Cell Carcinoma, Grade 2' and 'Adenocarcinoma, Pancreas, Grade II' (κ = 0.63) as well as 'Acute Lymphoblastic Leukemia Peripheral Blood, Grade Pre-B' and 'Acute Lymphoblastic Leukemia Bone Marrow, Grade Common' (κ = 0.61) (figure 3).

## Discussion

Despite comprehensive gene expression sets available on multiple human tumor entities, only few studies have attempted to assess and compare biological behavior of the tumors on the basis of functional microarray analysis and evaluation of commonly deregulated and conserved genetic signaling and metabolic events. Moreover, previous analyses focused on individual mechanisms or did not compose tumor-specific gene expression information into detailed, structured maps, impeding a systems view on cancer. However, in particular this kind of information could potentially guide medical research in the context of new applications and novel combinations of existing chemotherapeutics. Therefore, we here present gene expression information of tumors according to pathway activity profiles deemed to better reflect biological behavior. Our analysis of multiple tumor entities using these activity profiles resulted in a clearly structured functional cancer map (figure 2). Note: Due to experimental variation, wrong annotation, and simplifying model assumptions, our pathway signature almost surely fails to identify all active cellular processes in the respective experiment/cancer type [32]. But this is not the goal of the present work, our goal is to identify and distinguish relevant features of multiple tumor tissues; we demonstrate that the proposed analysis is sensitive enough for this purpose.

### Exposing functional mechanisms

As a proof of principle and supporting our approach, we found well documented biological events such as cell cycle and apoptosis to be highly conserved among the diverse tumor entities. These findings confirmed the reliability of the generated functional cancer map. Deregulated balance between proliferation and cell death represents a pro-tumorigenic mechansim in human carcinogenesis [33]. Both activation of proliferative signals and an inhibition of death process, leading to survival and consecutively to proliferation, may contribute essential steps in the progression to cancer in affected cells. Among highly enriched pathways in multiple tumors were 'Cell cycle', 'Signaling Molecules and Interactions', and 'Cell Growth and Death' summarizing genes involved in cell proliferation and apoptosis, demonstrating that key tumorigenic features of tumor development were identified by our analysis. Furthermore multiple functional pathways within the categories, 'Immune System' and 'Immune System Diseases' summarizing genes involved in immune response and immunological changes were also highly enriched in almost all tumor entities. Changes in these processes have been extensively documented to be involved in cancer development and growth [34-38]. Whenever normal body cells turn into tumor cells, various antigens on their surface change and were constantly secreted into the circulatory system. The so called tumor antigens were recognized by the immune system and cytotoxic T cells, natural killer cells and macrophages were advised to catch and eliminated the cell that undergo malignant transformation. A disturbed immune surveillance may lead to an increased tumor growth [34,38].

However, besides extensively studied and repeatedly documented signaling pathways highlighted in our analysis several pathways were found to be commonly enriched among differentially regulated genes which have not been clearly attributed to tumorigenesis. Thus, our work identified key roles of additional signaling pathways in cancer biology. Deregulations or mutations in the molecules involved in the 'Cell Cycle' and 'ECM-receptor interaction' pathway, preserving the physical link between extracellular matrix and actin cytoskeleton, are also closely connected to tumor progression [39]. Besides the 'Cell Cycle' pathway, the 'ECM-receptor interaction' pathway interacts with the 'Cell adhesion molecules (CAMs)'. This balance between cell adhesion and extra cellular molecules is essential for normal cell survival, imbalance between those pathways leads to detaching cells from the extra cellular matrix and therefore enhance metastasis [40,41]. However, the ECM-receptor interaction and mechanisms of focal adhesion were not in the focus of biomedical research on cancer mechanisms. Over the past decades, the majority of cancer related genetic studies were focussed on genes involved in transcription and cell cycle control. This is reflected in a tenfold higher number of abstracts containing the search terms 'cancer and cell cycle' than 'cancer and extracellular matrix or microenvironment' (122 594 vs. 20 287 articles) when entered in a PubMed query. This may be due to the fact that the plethora of biological functions of ECM proteins has been underestimated for a long time. For a long time functional properties of the extracellular microenvironment and matrix was believed to be restricted to form the "glue" between cells, responsible for simply to maintain shape and coherence of tissues and organs as well as a reservoir of body fluids. In the last decade the microenvironment gained increasing attention for many other processes including tumorigenesis [42] e.g. the breast microenvironment plays an important and complex role in hepatocarciongenesis. Therefore, cancers are currently regarded as heterogeneous multicellular entities containing cells of multiple lineages who interact with one another, the extracellular matrix (ECM), and soluble molecules in their vicinity are dynamic. Overall, these interactions favour cancer cell proliferation, movement, differentiation, and ECM metabolism, while simultaneously restricting cell death, stationary polarized growth, and ECM stability [43]. However, the microenvironment is also essential to key processes such as angiogenesis. Another important factor is the appreciation that cell migration, matrix and tissue remodeling are not unique properties of cancer cell growths but instead are tightly regulated programs normally utilized during development and in adult tissues responding to acute tissue injury [43]. Reciprocal interactions between responding "normal" cells, their mediators, structural components of the ECM, and genetically altered neoplastic cells regulate all aspects of tumourigenicity. An additional role was suggested for complex interaction of the extra cellular tumour stroma with (mesenchymal) stem cells involved in cancer growth [44]. Thus, demonstrating that functional pathways involved in ECM formation and loosening of focal adhesions are differentially regulated in almost all cancer entities, our results clearly advocate for enhanced efforts in unravelling molecular mechanisms of cancer development influenced by these pathways.

Almost no studies were published on the functional pathway of 'Complement and Coagulation cascades'. Few studies demonstrated a potential role of individual genes within this pathway in cancer development. Some members of the complement system can be modulated by the antifibrinolytic protein, activated thrombin-activatable fibrinolysis inhibitor (*TAFIa*), by inactivating the anaphylatoxins C3a, C4a and C5a and dysregulation of those genes has been implicated in tumor growth [45,46]. In addition a link between the haemostatic system and both tumor stroma and metastasis has been noticed in animal and in vitro models. The relationship between cancer and blood coagulation has been described on a diverse pathophysiological level. Cancer development and growth was shown to go along with hypercoagulable state and predisposes to activation of platelets and thrombosis, blood coagulation and fibrinolysis interfering with tumor cell biology, tumor growth, angiogenesis and metastatic process. However, the detailed molecular mechanisms of anti-coagulants such as warfarin on cancer remain elusive [47,48]. Given the strong enrichment of differentially regulated genes linked to the functional pathway of complement and coagulation, our data suggested that it may be worth to further elucidate exact underlying molecular mechanisms as they may hold key regulatory mechanisms in cancer development and growth.

Another promising candidate for further evaluation was the highly conserved PPAR signaling pathway. It is known that the peroxisome proliferation-activated receptor (PPAR) signaling pathway regulates a multitude of genes important for diverse cellular functions including cell proliferation, cell differentiation, immune response and apoptosis. Once activated, PPARgamma will preferentially bind with retinoid X receptor alpha and signal antiproliferative, antiangiogenic, and prodifferentiation pathways in several tissue types. Since ligands and other agents influencing this pathway have revealed anticancer effects in a variety of human cancers [49,50] efforts to target this signaling pathway should certainly be intensified given the broad range of tumors with deregulated PPAR signaling.

Finally, we noticed an enrichment of genes invovlved in the functional pathways "Diabetes type I" and "gluycolysis/gluconeogesis". Besides our data several clinical reports support a synergism between Diabetes mellitus and cancer development. Konishi I et al. reported a correlation between a diabetic pattern of the 75 g oral glucose tolerance test OGTT and hepatocarcinogenesis and considered it a significant risk factor for hepatocellular carcinoma (HCC) in patients with hepatitis C virus [51]. Also Huo et al. found that Diabetes mellitus independently predicts decreased survival in HCC patients [52]. Finally, El-Serag et al. reported that Diabetes mellitus is associated with an increased risk for HCC [53]. Interestingly the PPARgamma activators are utilized for the treatment of diabetes mellitus pointing towards a congruent signaling in of PPAR pathway and diabetes signatures [50].

In summary, there is accumulating evidence for a synergy between diabetes mellitus and an increased risk for and decreased survival of patients with HCC. Our data suggests that important interactions may be identified by analyzing the enriched functional pathways of "Diabetes type I" and "gluycolysis/gluconeogesis" previously not extensively studied in context of cancer.

### Exposing tumor phylogeny

We introduced a measure of profile similarity by which tumor entities were structured based on semantic aggregated profiles, which means significant, individual tumor mechanisms calculated out of gene expression data, into a dendrogram (figure 2). In order to get a measurement for genetic distance and thus to estimate the affinity between the diverse tumor entities, we applied the Cohen's Kappa Coefficient [21] to our binarized functional cancer map and clustered the distant relation matrix (figure 3). Predominant clustering of the tumor entities to their corresponding tumor class is indication for the robustness of the tumor phylogeny.

Notably, examining downregulated genes and clustering of the tumor entities by means of KEGG pathways, we found our sample to almost perfectly cluster according to the germ layer of the tissue of tumor origin. However, this finding needs to be further analyzed and applied to robust statistical testing [54,55].

## Conclusion

Multiple tumor entities have been analyzed for signaling and metabolic events in tumor progression and composed into a functional cancer map. The resulting detailed, structured map should lead to a more systematic view on common and individual tumor mechanisms. This work lead to the identification of novel superimposed functional pathways closely linked to cancer biology. Among these pathways were 'ECM-receptor interaction', 'Complement and Coagulation cascades', and 'PPAR signaling pathway'. Furthermore tumor phylogeny was calculated with respect to their biological behavior. Both, the functional cancer map paired with the indication for tumor phylogeny could serve as a starting point for the efficient combination of targeted tumor therapeutics as well as for expanding the application area of well-established tumor therapies.

## Competing interests

The authors declare that they have no competing interests.

## Authors' contributions

Primary data preparation and normalization: TB, RK; Data analysis: MK, TM, JM, PRG, AT; Statistics and pathway analsis: FS, SB, ATr; Manuscript preparation: MK, JM, ATr, AT. All authors have read and approved the final manuscript.

## Pre-publication history

The pre-publication history for this paper can be accessed here:

http://www.biomedcentral.com/1755-8794/4/53/prepub

## Supplementary Material

Additional file 1 **List of regulated tumor genes with respect to tumor entities and corresponding p-values / q-values**. Click here for file

Additional file 2 **Functional cancer map with respect to upregulated genes**. Click here for file

Additional file 3 **Functional cancer map with respect to downregulated genes**. Click here for file

Additional file 4 **Functional cancer map without any filtering for outlier genes**. Click here for file

Additional file 5 **PubMatrix result for the literature mining of the enriched KEGG pathway maps in context of published literature to tumor progression**. Click here for file

Additional file 6 **Tumor phylogeny: Calculation of the tumor phylogeny was done by applying the Cohen's Kappa Coefficient to the binarized upregulated functional cancer map**. Click here for file

Additional file 7 **Tumor phylogeny: Calculation of the tumor phylogeny was done by applying the Cohen's Kappa Coefficient to the binarized downregulated functional cancer map**. Click here for file
