# Supplementary figures and images for: The functional cancer map: A systems-level synopsis of genetic deregulation in cancer
Source: BMC Med Genomics. 2011 Jun 30;4:53. doi: 10.1186/1755-8794-4-53 (PMC3148554; doi:10.1186/1755-8794-4-53)

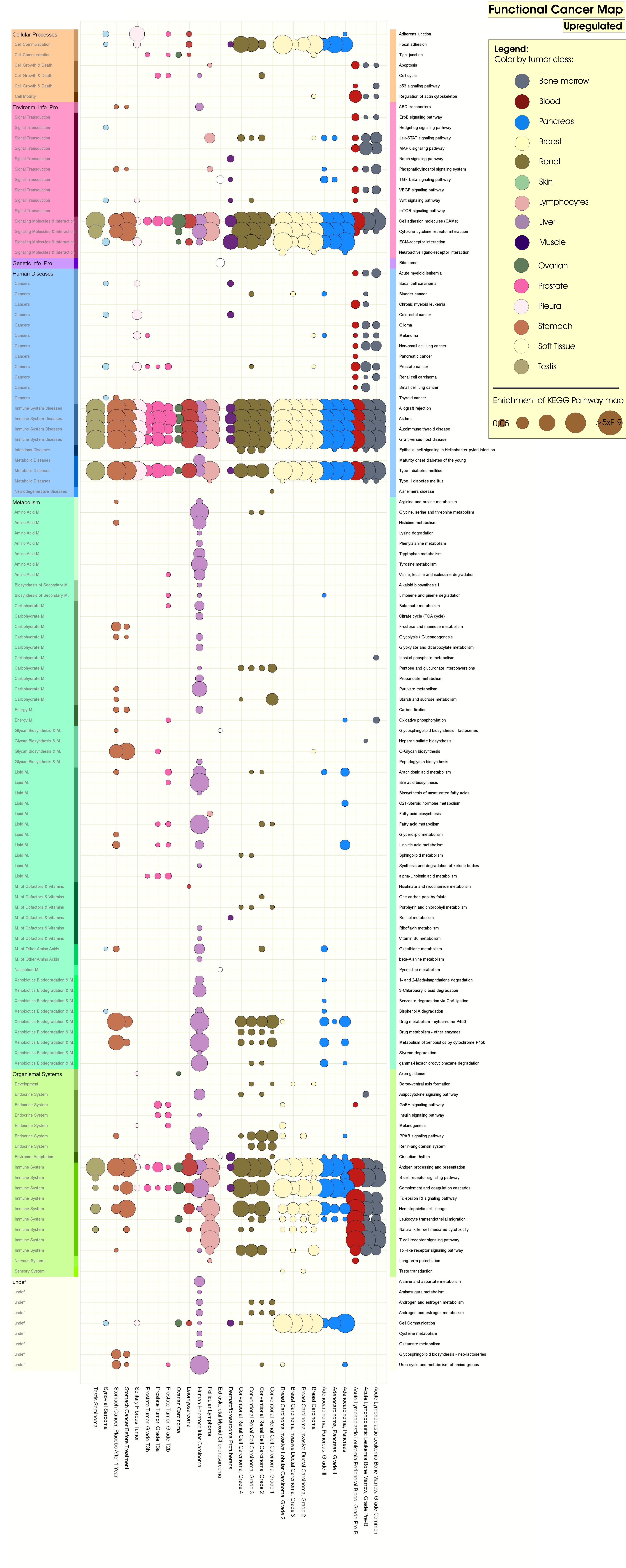

Supplement: Additional file 2 — Functional cancer map with respect to upregulated genes. [file 1755-8794-4-53-S2.PNG]

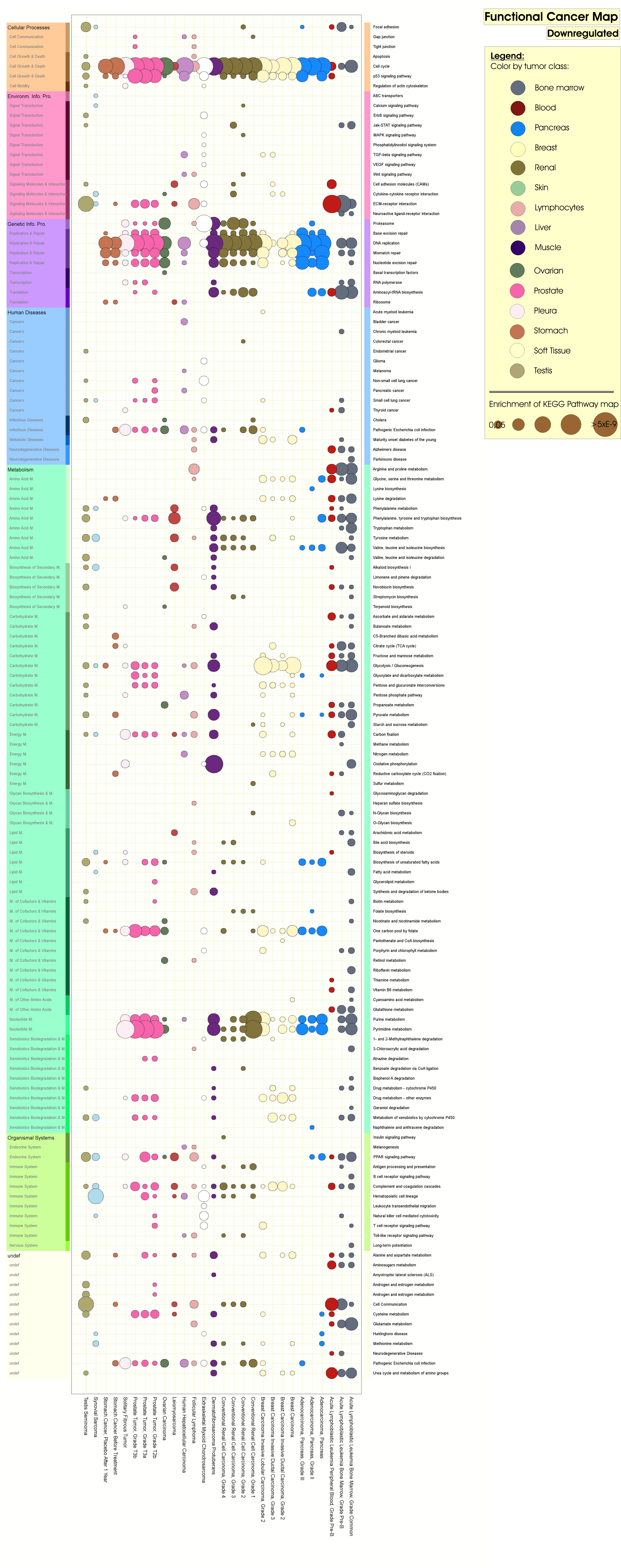

Supplement: Additional file 3 — Functional cancer map with respect to downregulated genes. [file 1755-8794-4-53-S3.PNG]

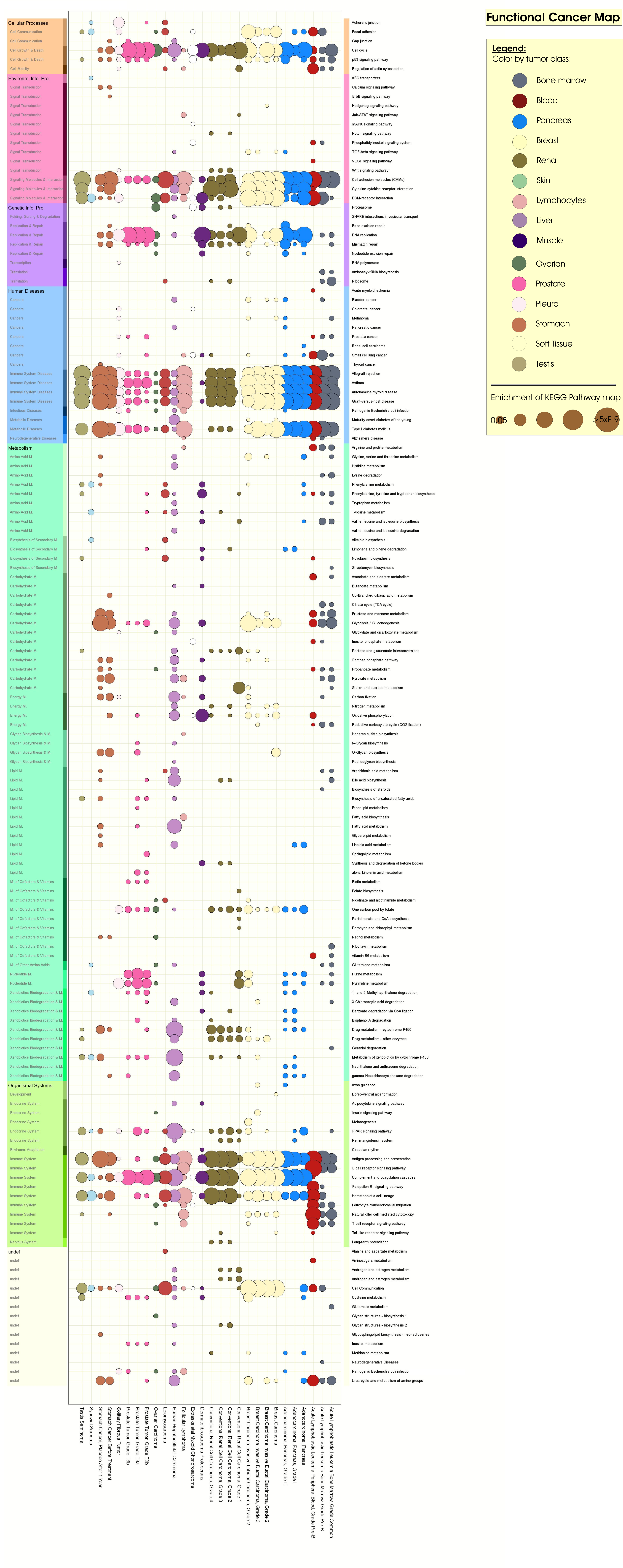

Supplement: Additional file 4 — Functional cancer map without any filtering for outlier genes. [file 1755-8794-4-53-S4.PNG]

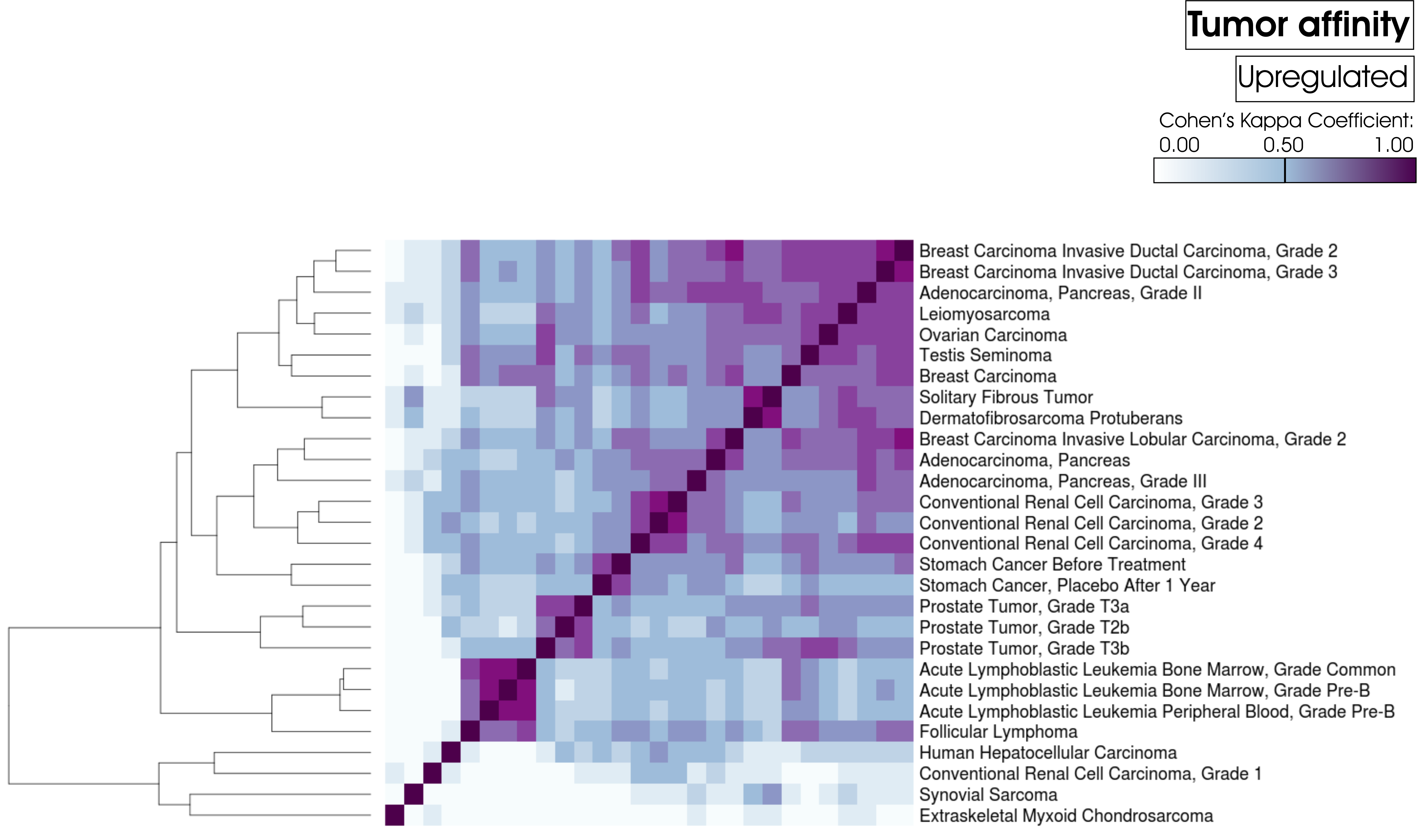

Supplement: Additional file 6 — Tumor phylogeny: Calculation of the tumor phylogeny was done by applying the Cohen's Kappa Coefficient to the binarized upregulated functional cancer map. [file 1755-8794-4-53-S6.PNG]

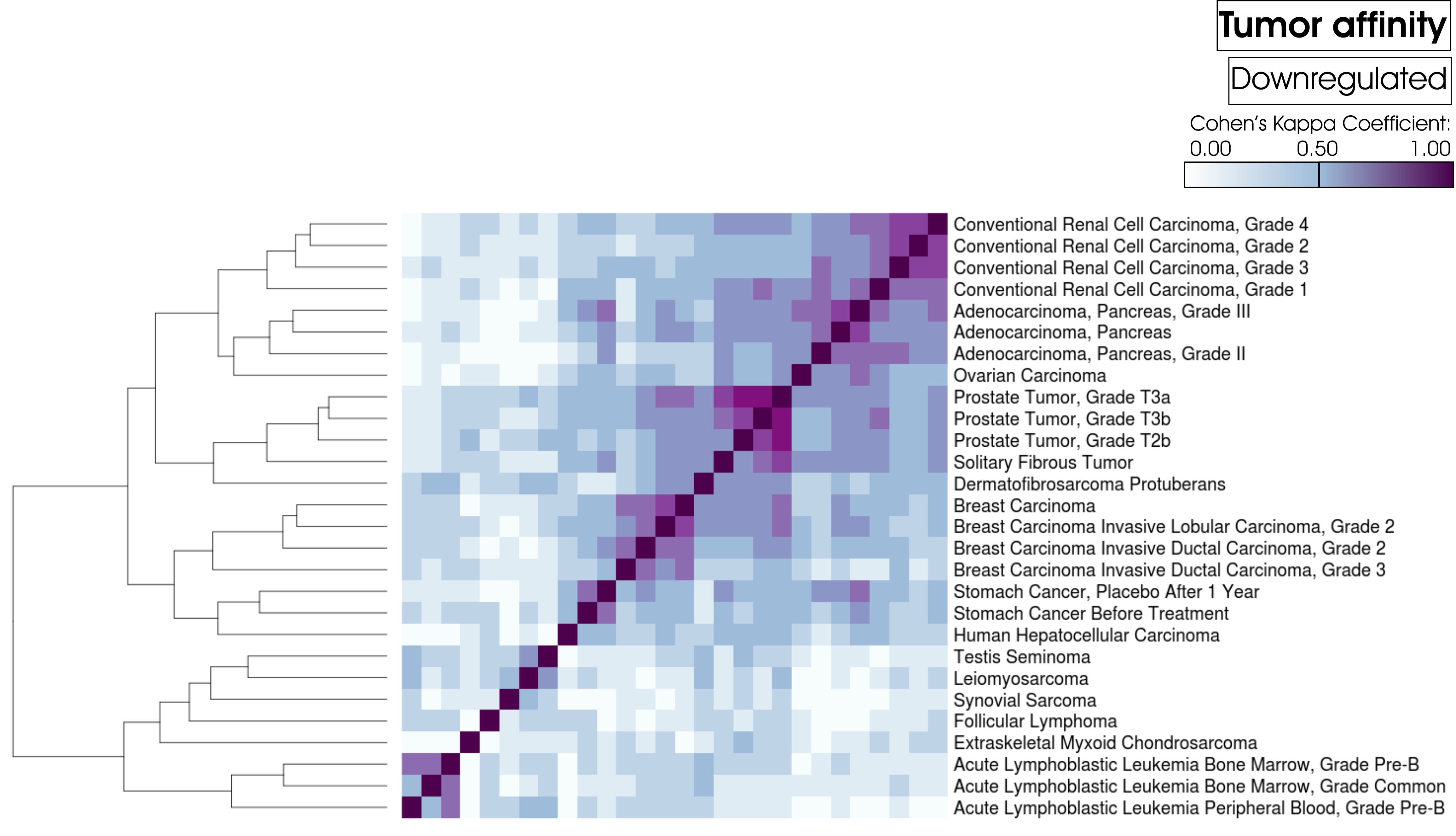

Supplement: Additional file 7 — Tumor phylogeny: Calculation of the tumor phylogeny was done by applying the Cohen's Kappa Coefficient to the binarized downregulated functional cancer map. [file 1755-8794-4-53-S7.PNG]
